# Upregulation of the WNK4 Signaling Pathway Inhibits Epithelial Sodium Channels of Mouse Tracheal Epithelial Cells After Influenza A Infection

**DOI:** 10.3389/fphar.2019.00012

**Published:** 2019-01-22

**Authors:** Yapeng Hou, Yong Cui, Zhiyu Zhou, Hongfei Liu, Honglei Zhang, Yan Ding, Hongguang Nie, Hong-Long Ji

**Affiliations:** ^1^Department of Stem Cells and Regenerative Medicine, Key Laboratory of Cell Biology, National Health Commission of China, Key Laboratory of Medical Cell Biology, Ministry of Education of China, China Medical University, Shenyang, China; ^2^Department of Anesthesiology, The First Affiliated Hospital of China Medical University, Shenyang, China; ^3^Department of Cellular and Molecular Biology, The University of Texas Health Science Center at Tyler, Tyler, TX, United States; ^4^Texas Lung Injury Institute, The University of Texas Health Northeast, Tyler, TX, United States

**Keywords:** ENaC, influenza virus, airway surface liquid, WNK4, MTECs

## Abstract

Influenza virus has a significant impact on the respiratory system. The mechanism of how influenza virus impairs the fluid transport in airway is not fully understood. We examined its effects on epithelial sodium channels (ENaC), which are very important for water and salt transport in the respiratory system. We focused on the impacts of influenza virus on ENaC activity in mouse tracheal epithelial cells (MTECs) and applied Ussing chamber apparatus for recording the short-circuit currents in primary cultured MTECs. Expressions of α and γ-ENaC were measured at the protein and mRNA levels by western blot and quantitative real-time polymerase chain reaction, respectively. Roles of the with-no-lysine-kinase-4 (WNK4) pathway were considered in participating influenza virus-involved ENaC regulation by using siRNA to knockdown WNK4 and the physical properties of airway surface liquid (ASL) were detected by confocal microscopy. Our results showed that influenza virus reduced ENaC activity, and the expressions of α and γ-ENaC were decreased at the protein and mRNA levels, respectively. WNK4 expression increased time-dependently at the protein level after influenza virus infection, while knockdown of WNK4 rescued the impact of influenza virus on ENaC and ASL height increased obviously after MTECs were treated with influenza virus. Taken together, these results suggest that influenza virus causes the changes of biophysical profile in the airway by altering the ENaC activity at least partly via facilitating the expression of WNK4.

## Introduction

Influenza is an infectious disease caused by influenza virus. There are three kinds of influenza viruses, A, B, and C, among which influenza virus A and B regularly infect humans and are responsible for influenza epidemics (Londino et al., 2017). The influenza A is a negative sense RNA virus that contains eight gene segments coding for approximately 13 proteins (Noah et al., 2009). Influenza virus A infections are always accompanied with a series of clinical symptoms, including cough, sore throat, lung edema, and even death (Peteranderl et al., 2016). Influenza is fatal to weak and chronic disease patients, especially for the elderly and children (Hilleman, 2002).

In normal respiratory tract, about 80% of the cells are epithelial cells with cilia. Mouse tracheal epithelial cells (MTECs) are mainly composed of the polarized columnar cells with a brush of cilia on the top (Ibricevic et al., 2006), and the polarized epithelial cells and extracellular fluid constitute the first physical barrier to the respiratory system for harmful substances such as virus and bacteria. ASL at least consists of mucus layer and preciliary liquid layer, the former of which contains highly glycosylated mucins rich in terminal sialic acid, and is thought to be able to trap influenza virus from the environment and eliminate them from respiration system by cilia beating in a mechanical manner (Knowles and Boucher, 2002; Lillehoj and Kim, 2002; Matrosovich and Klenk, 2003; Duez et al., 2009; Barbier et al., 2012). The height of ASL is a common index reflecting the ability of fluid transport under pathological conditions.

Epithelial sodium channels (ENaC) are the main pathway and the rate-limiting step for Na^+^ to transfer from the apical to the basolateral side of epithelial cells with high selectivity (Eaton et al., 2014; Kellenberger and Schild, 2015; Joo et al., 2016). The apical membranes of many tight epithelia contain ENaC that are characterized primarily by their high affinity for the diuretic blocker amiloride (Hanukoglu and Hanukoglu, 2016). To date, five subunits of ENaC have been cloned, named α, β, γ, δ and ε-ENaC, the first four of which are expressed in human. ENaC are distributed in the lungs, kidneys and other organs, facilitating Na^+^ reabsorption across the apical membranes of epithelia in the respiratory, distal nephron and exocrine glands. Several diseases are relevant to ENaC, such as asthma and lung edema. In the respiratory tract, ENaC are regulators of the osmolarity of ASL, which are essential to maintain fluid volume at a depth necessary for cilia beating (Enuka et al., 2012; Eaton et al., 2014).

Many factors can modulate ENaC, such as aldosterone, growth factors, serine proteases, and protein kinases, among which with-no-lysine-kinase-4 (WNK4) had been reported to inhibit ENaC *via* a mechanism that is independent of its kinase activity. This inhibition requires intact C termini in ENaC β- and γ- subunits, which contain PY motifs used to target ENaC for clearance from the plasma membrane (Ring et al., 2007). It was assumed that WNK4 could inhibit ENaC activity by reducing the total amount of cellular ENaC as well as ENaC at the apical membrane, but it had no effect on channel open probability. The net reduction in ENaC surface expression could be due to either WNK4 enhancing the rate of ENaC internalization or reducing its delivery to the apical membrane or a combination of both, and these inhibition effects are independent of Nedd-4-2 mediated ENaC ubiquitination (Yu et al., 2013). However, how to modulate the ENaC-related airway fluid regulation under influenza infection is not fully understood.

## Materials and Methods

### Virus

The influenza virus A/PR/8/34 (PR8, H1N1, purchased from China Center for Type Culture Collection, China) was injected to allantoic cavity of 10-day chicken embryo. After 2 days at 35°C, the allantoic fluid was harvested and centrifuged at 900 *g* for 5 min, then stored at −70°C. Infectivity of influenza virus was titered on the monolayers of Madin-Darby canine kidney cells.

### MTEC Culture

The healthy male C57 mice were provided by the Laboratory Animal Center of China Medical University. All experiments involving C57 mice were performed according to the guidelines and regulations of Animal Care and Use Ethics Committee and all experimental protocols were approved by China Medical University. Tracheal was removed from diazepam (17.5 mg kg^−1^, intraperitoneally) followed 6 min later by ketamine (450 mg kg^−1^, intraperitoneally) anesthetized mouse, and digested with 3 ml 0.1% protease XIV, 0.01% DNA enzyme and 1% FBS in DMEM for 24 h at 4°C. 1 ml FBS was added to stop digestion, and after centrifugated twice at room temperature, cells were resuspended immediately in complete medium. 0.5 ml complete medium was added to each well outside of the insert, and cells were seeded onto 6.5-mm diameter mouse tail collagen I pre-coated Transwell inserts (Corning-Costar, Lowell, MA, United States) at a density of 1.5 × 10^5^ cells/cm^2^. MTEC complete medium consists of 1:1 mixture of 3T3 fibroblast preconditioned DMEM (containing 4 mM L-Glutamine, 4500 mg/L Glucose, 10% FBS, 1% penicillin/streptomycin) and Ham’s F-12 medium (containing 1 mM L-Glutamine), supplemented with 10 μg/ml insulin (Gibco, New York, NY, United States), 1 μM hydrocortisone (Sigma, St. Louis, MO, United States), 250 nM dexamethasone (Sigma, St. Louis, MO, United States), 3.75 μg/ml bovine endothelial cell growth supplement (Cell Applications, Inc., San Diego, CA, United States), 25 ng/ml epidermal growth factor (Sigma, St. Louis, MO, United States), 10 ng/ml cholera toxin (Macgene, China), 30 nM triiodothyronine (Sigma, St. Louis, MO, United States), 5 μg/ml iron saturated transferring (Gibco, New York, NY, United States) and 30 μg/ml bovine pituitary extract (SceinCell, San Diego, CA, United States). 4 days after seeding, apical fluid was removed to establish air–liquid interface and the medium was changed every other day. Trans-epithelial electrical resistance was monitored when culture medium was replaced using an epithelial voltohmmeter (WPI, Sarasota, FL, United States). Cell-growing inserts with a resistance >1000 Ω cm^2^ were used. 1.2 × 10^5^ PFU influenza virus was diluted in PBS and added to the apical surface of cells at a MOI of 0.6. The final volume of virus diluent was 100 μl, while the control group was treated with isovolumetric PBS. Virus was removed from the top medium 1 h post-infection at 37°C and all the following experiments were done after 24 h.

### Ussing Chamber Assay

Measurements of trans-epithelial electrical resistance and Isc were performed as previously described (Li et al., 2017). MTEC monolayers were mounted in Ussing chambers (Physiologic Instruments, San Diego, CA, United States) and bathed on both sides with a solution contained (in mM) 120 NaCl, 25 NaHCO_3_, 3.3 KH_2_PO_4_, 0.83 K_2_HPO_4_, 1.2 CaCl_2_, 1.2 MgCl_2_, and 10 HEPES, supplemented with either 10 mannitol (apical compartment) or 10 glucose (basolateral compartment). The osmolality of each solution was between 290 and 300 mOsm/kg. Both sides of the bath solutions (pH 7.4) were bubbled with 95% O_2_ and 5% CO_2_ continuously at 37°C. The Isc levels were measured with 3 M KCl, connected on either side to Ag–AgCl electrodes filled with 4% agarose salt bridges. MTEC monolayers were short-circuited to 0 mV, and a 10 mV pulse of 1 s was given every 10 s to monitor trans-epithelial electrical resistance. When the Isc was stable, 10 μl ENaC specific inhibitor amiloride (50 mM stock) was pipetted into 5 ml apical bath solution, and the final concentration of amiloride was 100 μM. Data were collected using the Acquire and Analyse program version 2.3.

### Transmission Electron Microscopy

For transmission electron microscopy, MTEC monolayers were fixed with 2% glutaraldehyde, 4% paraformaldehyde in 0.1 M Na-Cacodylate buffer for 15 min at room temperature. Then they were stained with 1% aqueous osmium tetroxide for 2 h, dehydrated by incubating at alcohol gradient, embedded with EMbed 812, and cut into 80–100 nm sections. The slides were finally stained with uranyl acetate and lead citrate, and analyzed by transmission electron microscope. The width of tight junction was determined from 5 sections of 10 randomly selected fields in each section for each sample (Cong et al., 2012).

### Western Blot Assays

The MTEC transwell insert was put on ice, and washed by cold PBS twice. Laemmli sample buffer was added to the transwell insert and transferred to a microcentrifuge tube on ice. After separation on SDS-PAGE (10% polyacrylamide gels), proteins were trans-blotted onto PVDF membranes (Invitrogen, Waltham, MA, United States). The membranes were blocked with 5% BSA in 1× TBST containing 20 mM Tris-HCl (pH 7.5), 0.5 M NaCl, 1% Tween at room temperature for 1 h, and then incubated with primary antibody at a dilution of 1:2,000 (α, γ-ENaC) or 1:500 (WNK4) in 5% BSA at 4°C overnight. Primary antibodies were used to detect ENaC subunits α-ENaC (PA1-920A, Thermo Fisher, Waltham, MA, United States), γ-ENaC (ab3468, Abcam, Cambridge, MA, United States), WNK4 (5713, Cell Signaling, Danvers, MA, United States) and β-actin (sc-47778, Santa Cruz Biotechnology, Santa Cruz, CA, United States). The membranes were washed six times with TBST for 5 min intervals before being incubated with horseradish peroxidase-conjugated goat-anti-rabbit or goat-anti-mouse secondary antibody at a dilution of 1:5,000 in 5% milk. The image was developed by ECL kit after washing membranes three times for 10 min intervals, and data were collected using the Image J program.

### Polymerase Chain Reaction (PCR)

Total RNA was extracted using TRIzol reagent (Invitrogen, Waltham, MA, United States), according to the manufacturer’s instructions, and RNA concentration was measured by spectrophotometry at 260 nm. The following primer pairs were used for real-time PCR: α-ENaC forward (5′-AAC AAA TCG GACTGC TTC TAC-3′) and reverse (5′-AGC CAC CAT CAT CCA TAA A-3′), β-ENaC forward (5′-GGG ACC AAA GCA CCA AT-3′) and reverse (5′-CAG ACG CAG GGA GTC ATAG-3′), γ-ENaC forward (5′-GCACCG TTC GCC ACC TTC TA-3′) and reverse (5′-AGG TCA CCA GCA GCT CCT CA-3′), and GAPDH forward (5′-AGA AGG CTG GGG CTC ATT TG-3′) and reverse (5′-AGG GGC CAT CCA CAG TCT TC-3′). The following primer pairs were used for reverse transcription PCR: WNK4 forward (5′-TGA TGC TAG ACT GGC ACC TAT ATC TGA-3′) and reverse (5′-TCC TTC TTC TGT AGT GTC TGT AGT GC-3′), and GAPDH forward (5′-TGC CTG CTT CAC CAC CTT CT-3′) and reverse (5′-AGG CCG GTG CTG AGT ATG TC-3′). Total RNA (1 μg) was used as a template for reverse transcription, and all samples were run in triplicate in a final volume of 20 μl. Reaction for ENaC subunits was performed using a single cycle of 95°C for 0.5 min, followed by 50 cycles of 95°C for 5 s, 52°C for 0.5 min, and 72°C for 1 min. PCR of WNK4 was performed in the following condition: single cycle of 95°C for 1 min, followed by 30 cycles of 95°C for 1 min, 59°C for 45 s, 72°C for 1 min, and a single cycle of 72°C for 10 min. 5 μl of PCR products were run on 1.2% agarose gel and visualized under the ultraviolet light. Relative expression of ENaC mRNA was calculated using the 2^−ΔΔCT)^ comparative method, with normalization of each sample against the expression of endogenous reference gene GAPDH.

### Measurements of ASL Height

Airway surface liquid was labeled with 10 kDa FITC-dextran (100 μl at 0.5 mg/ml in PBS, administered 24 h prior to measurements). Transwell membranes were placed in a glass bottom dish covered with 150 μl high-boiling-point perfluorocarbon octadecafluorooctane (Yiji Industrial Company, China) to prevent evaporation. ASL height was measured by laser z-scanning microscopy, using a Nikon C2 confocal microscope (Nikon, Tokyo, Japan) equipped with a 40 oil-immersion objective. To determine average fluid height, there are five predetermined points, one central and four 2 mm from the edge of the culture. During the recording, the perfusion chamber was maintained at 37°C in an incubator and optical section was obtained within 10 min.

### Fluorescence Recovery After Photobleaching

The experiment condition of photobleaching was the same with ASL height measurement. Photobleaching was done in the fluorescently labeled mucus layer (2 μm below the upper surface). The fluorescence intensity of a circular region was acquired for 60 s before photobleaching, then the laser power was changed to reduce fluorescence by 30–45% in the circular bleach zone compared with the intensity before the photobleaching. The recovery fluorescence intensity was acquired during the next 3 min and the 30% recovery time was determined that after the fluorescence intensity is decreased to the lowest point, the time it takes from the minimum to recover 30% of the decreased fluorescence intensity. All photobleaching measurements were done within 10 min.

### ELISA Assays

The concentration of WNK4 was detected according to the manufacturer’s instructions (Jinyibai Biotech Company, China). After 12, 24, and 48 h post-infection to influenza virus respectively, MTECs were freeze-thawed repeatedly for three times. Damaged cells and released intracellular components were removed and the supernatant was collected for measurement.

### Knockdown of WNK4

Hundred μl mixture of siRNA for WNK4 or negative control (GenePharma, China) and siRNA-mate were added to the apical side of MTECs according to the manufacturer’s instructions. Two kinds of siRNA, named WNK4-1816 and WNK4-3088, were provided. The following sequence were used for WNK4 knockdown experiment: negative control forward 5′-UUC UCC GAA CGU GUC ACG UTT-3′ and reverse 5′-ACG UGA CAC GUU CGG AGA ATT-3′, WNK4-1816 forward 5′-UAG UUG AUG AGU AGC UGG CTT-3′ and reverse 5′-GCC AGC UAC UCA UCA ACU ATT-3′, WNK4-3088 forward 5′-GGC CFU UUC CAA GUG SCU UTT-3′ and reverse 5′-AAG UCA CUU GGA AAC GGC CTT-3′. The final concentration of siRNA was 200 nM and the transfection reagent was removed after 6 h. ENaC functional analysis was performed after 72 h post-transfection.

### Statistical Analysis

All results were presented as mean ± SE. ENaC activity is the difference of the total and amiloride-resistant current fractions. Normality and homoscedasticity test was done by Levene and Shapiro–Wilk test before applying parametric tests. For comparison of two groups, we used Student’s two-tailed *t*-test; for comparison of multiple groups, we performed one-way analyze of variance (ANOVA) followed by Bonferroni’s test for all the groups of the experiment. When the data did not pass the normality or homoscedasticity test, we used a non-parametric *t*-test (Mann–Whitney *U*-test). Variations were considered significant when the *P*-value was less than 0.05. Statistical analysis was performed with Origin 8.0.

## Results

### Influenza Virus Reduced Amiloride-Sensitive Isc

**FIGURE 1:**
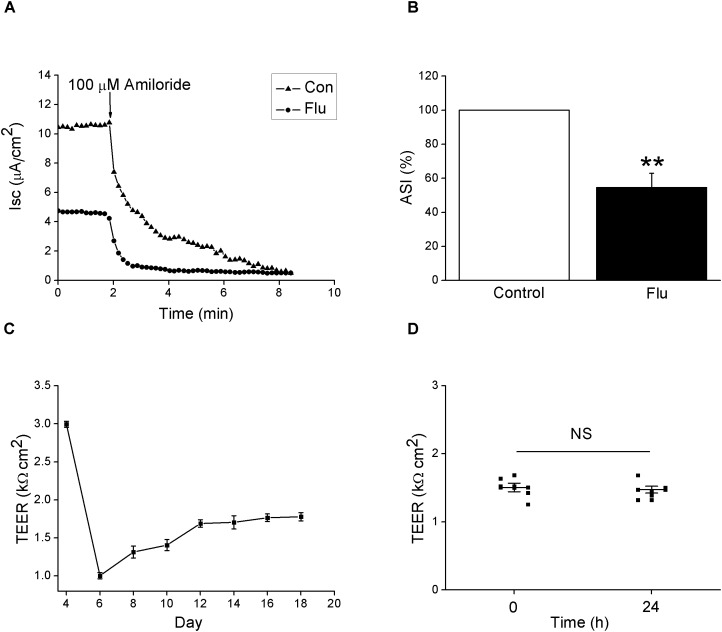
Amiloride-sensitive short-circuit currents in MTEC monolayers was reduced by influenza virus. **(A)** Isc trace after MTECs were infected with influenza virus (Flu) at MOI 0.6 for 24 h, then amiloride was applied. **(B)** Statistic ASI in MTEC monolayers. ASI was defined as the difference between the total current and the amiloride-resistant current and the initial ASI was set as 100%. ^∗∗^*P* < 0.01, compared with control (Con), *n* = 5. Data was presented as mean ± SE Mann–Whitney *U*-test was used to analyze the difference of the means for significance. **(C)** Development of trans-epithelial electrical resistance (TEER) during MTEC culturing. *n* = 5. **(D)** Changes in TEER after treated with influenza virus. NS, *P* > 0.05, *n* = 6. Student’s *t*-test was used to analyze the difference of the means for significance.

Epithelial sodium channels are responsible for salt-water transport in airway, which can affect ASL composition, height, and mucus-ciliary clearance (Enuka et al., 2012). We hypothesized that ENaC are the targets for the change of the biophysical properties caused by influenza virus in airway. First we testified the effect of influenza virus on the transport of Na^+^ by measuring amiloride-sensitive Isc (ASI) in Ussing chamber system. MTEC is an ideal primary cell model for the study of respiratory diseases, which basically accords with the morphological and physiological characteristics of the airway *in vivo* (Davidson et al., 2000; Horani et al., 2013). The results showed that influenza virus infection reduced Isc, which could be blocked by 100 μM amiloride, a specific ENaC inhibitor (Figure 1A). We defined ASI as the difference between the total current and the amiloride-resistant current and set the initial ASI as 100%. As shown in Figure 1B, after MTEC monolayers were infected with influenza virus for 24 h, the ASI significantly decreased (*P* < 0.01 versus Control, *n* = 5). We also monitored the development of trans-epithelial electrical resistance and possible difference in resistance after infection when the culture medium was replaced every other day. The trans-epithelial electrical resistance was highest early in air–liquid interface and showed nearly stable after MTEC were cultured 12 days in transwells (Figure 1C). Influenza virus infection did not change the electrical resistance obviously within 24 h (Figure 1D). Overall, these results suggest that influenza virus can inhibit the activity of ENaC.

**FIGURE 2:**
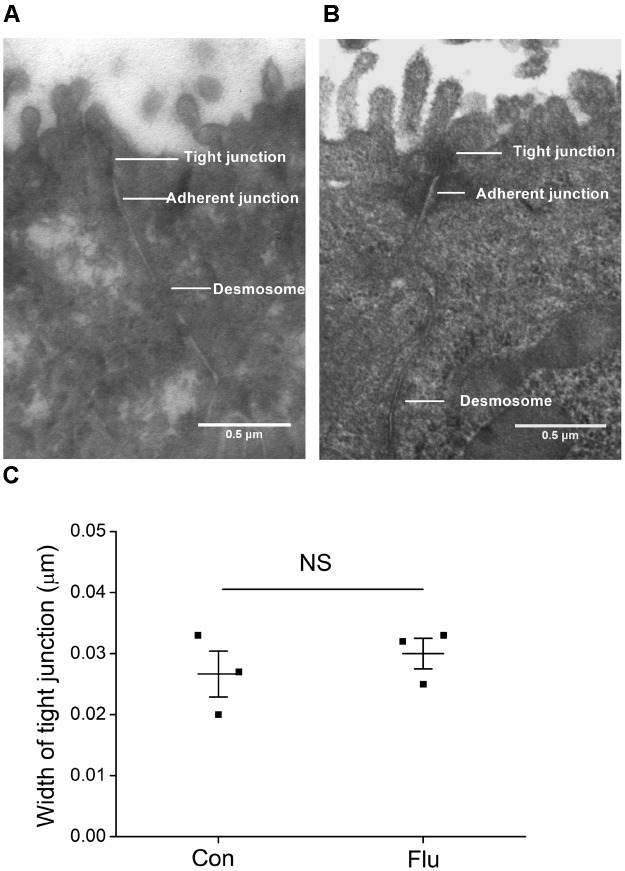
Ultrastructure in MTECs by transmission electron microscope. **(A)** Tight junctions in uninfected MTECs (Con), which include tight junctions, adherent junctions and desmosome. **(B)** Tight junctions in influenza virus infected (Flu) MTECs. **(C)** Tight junction width in MTECs, NS, *P* > 0.05, *n* = 3. Data was presented as mean ± SE Student’s *t*-test was used to analyze the difference of the means for significance.

In order to remove the possibly disturbing effects of the influenza virus on ASI by destroying the tight junctions, we applied the transmission electron microscopy to detect the structure of tight and adherent junctions in MTECs. As shown in Figures 2A–C, there is no significant difference of the width of tight junctions between the control and the influenza virus infection group (*P* > 0.05, *n* = 3), suggesting that the tight junctions in MTECs were not destroyed obviously by influenza virus within 24 h (Wu et al., 2016).

### Expressions of α and γ-ENaC Were Decreased at Protein and mRNA Levels by Influenza Virus

**FIGURE 3:**
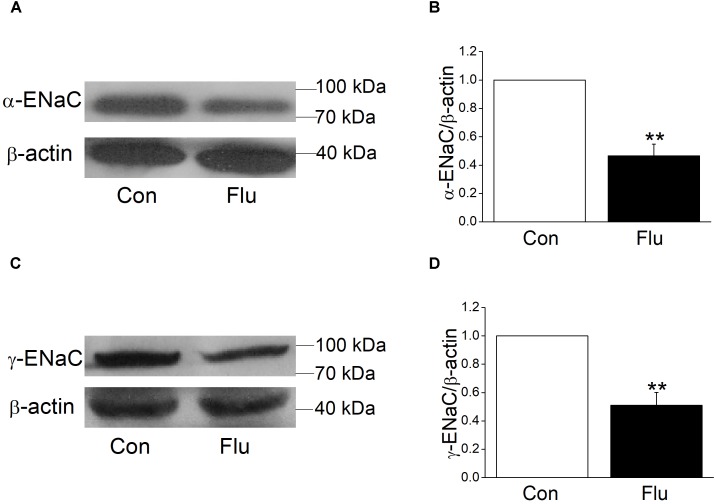
Effects of influenza on the protein expression level of α- and γ-ENaC subunits in MTECs. MTECs were infected to influenza virus (Flu) for 24 h and then proteins were extracted and analyzed by western blot. **(A,C)** Western blot bands of α- and γ-ENaC subunits demonstrating reductions in protein levels. Blots for β-actin were used as internal controls. **(B,D)** Graphical representation of data obtained from western blot assays for α- and γ-ENaC subunits, which were quantified using gray analysis (α-ENaC/β-actin and γ-ENaC/β-actin), ^∗∗^*P* < 0.01, compared with control (Con), *n* = 3–4. Data was presented as mean ± SE Student’s *t*-test was used to analyze the difference of the means for significance.

Mouse tracheal epithelial cells were infected with influenza virus for 24 h and the protein expression levels were observed by western blot, with β-actin set as an internal standard. The specific band about 80 kDa for α-ENaC protein could be seen, according to the manufacturer’s manual and previous studies (Rahman et al., 2010). The α-ENaC antibody they used was from Affinity Bio Reagents, which was taken over by Thermo Fisher (we bought the antibody from) and the same Catalog Number was kept. The specific band about 95 kDa for γ-ENaC protein could be detected, according to the manufacturer’s manual. We also tested the specificity of these antibodies in the additional experiment. As shown in Supplementary Figure 1, co-incubation of these anti-ENaC antibodies with an excess of corresponding immunizing peptides prevented almost completely the appearance of the bands. Our results showed that influenza virus could reduce the expression of α- and γ-ENaC protein (Figures 3A–D, *P* < 0.01 versus Control, *n* = 3–4). We didn’t detect the β-ENaC expression for the lack of suitable antibody for the western blot assay.

**FIGURE 4:**
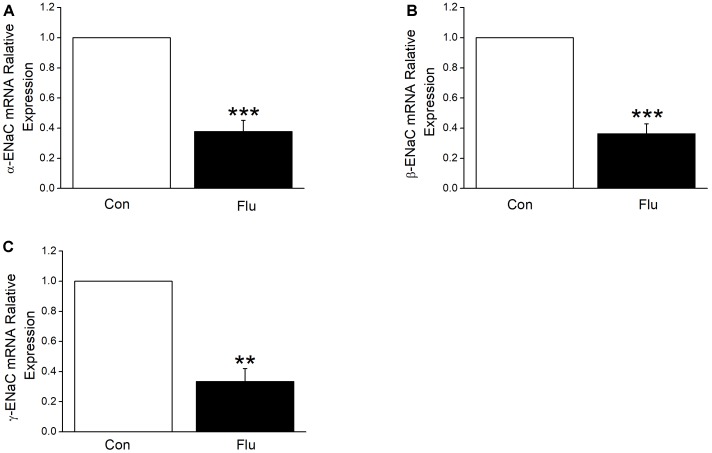
Influenza virus decreased the transcriptional expression of ENaC. The mRNA samples were isolated from MTECs infected with influenza virus (Flu) for 24 h. Relative levels of mRNA were calculated as α- or β- or γ-ENaC/GAPDH ratios. **(A–C)** Quantitive-real-time PCR results for α-, β-, and γ-ENaC mRNAs. ^∗∗^*P* < 0.01, ^∗∗∗^*P* < 0.001, compared with control (Con), *n* = 4–5. Data was presented as mean ± SE Student’s *t*-test was used to analyze the difference of the means for significance in **(A,B)**, and Mann–Whitney test was used to analyze the difference of the means for significance in **(C)**.

Based on the results of influenza virus reduction in the expression of ENaC protein, we suspected that influenza virus may also reduce ENaC transcription levels, which were the former step in protein synthesis. We tried quantitive-real-time PCR to determine the mRNA expression levels of the three subunits of ENaC accordingly and as expected, when MTECs were infected by influenza virus for 24 h, α, β, and γ-ENaC mRNA were all decreased significantly (Figures 4A–C, *P* < 0.001 ∼ 0.01 versus Control, *n* = 4–5), proving that influenza virus can significantly reduce ENaC expression at mRNA transcription levels.

**FIGURE 5:**
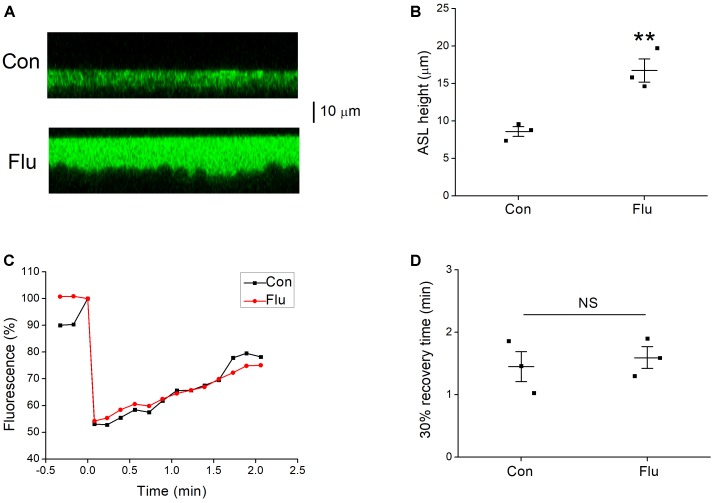
Effects of influenza virus on ASL height. **(A)** Images from confocal microscopy, the ASL (green) were covered with perfluorocarbon Octadecafluorooctane. **(B)** Mean data of ASL height for control (Con) and influenza virus (Flu) infected MTECs. ^∗∗^*P* < 0.01, compared with control, *n* = 3. **(C)** Representative fluorescence recovery curve of photobleaching. **(D)** 30% recovery time, which was determined that after the fluorescence intensity is decreased to the lowest point, the time it takes from the minimum to recover 30% of the decreased fluorescence intensity. NS, *P* > 0.05, *n* = 3. Data was presented as mean ± SE Student’s *t*-test was used to analyze the difference of the means for significance.

### Influenza Viruses Increased ASL Height

Active Na^+^ and Cl^−^ transport are essential for maintaining homeostasis of the ASL (Tarran et al., 2006). The impact of influenza virus on ENaC function results in disorders of Na^+^ transport, and we speculated that the biophysical properties of ASL could also be changed. The confocal microscopy was used to determine the ASL height, and the results showed that the ASL height of MTECs in the influenza virus infection group was much higher than that of control group (Figures 5A,B, *P* < 0.01, *n* = 3). The phenomenon that influenza virus increases the ASL height, indicates that the volume of the ASL could also be changed (Tarran and Boucher, 2002), while mucus clearance rate, cilia beating mode, cilia beating frequency, and a series of respiratory epithelial biophysical properties altered accordingly (Liu et al., 2013, 2014), all of which aid influenza virus to damage the first line of defense and increase the susceptibility to other pathogens.

The homeostasis of ASL may also be destroyed for the change of the ASL height. We speculated that influenza virus may change the viscosity of mucus layer, which has a greater impact on the velocity of mucociliary transport compared with cilia beat frequency (Sedaghat et al., 2016), and we applied confocal microscopy to detect the viscosity of mucus layer in MTECs. Fluorescence recovery after photobleaching is a technique for measuring the viscosity of mucus layer (Jayaraman et al., 2001; Derichs et al., 2011), but unfortunately, we found that influenza virus had nearly no effect on the typical fluorescence recovery curve of mucus layer (Figure 5C), which decreased to about 55% in Fluorescence after photobleaching. The 30% recovery time was determined that after the fluorescence intensity is decreased to the lowest point, the time it takes from the minimum to recover 30% of the decreased fluorescence intensity, and as shown in Figure 5D, the 30% recovery time had no difference between the control and influenza virus infection group (*P* > 0.05, *n* = 3), which means that influenza virus does little change on the viscosity of mucus layer.

### Influenza Viruses Increased the Expression of WNK4

**FIGURE 6:**
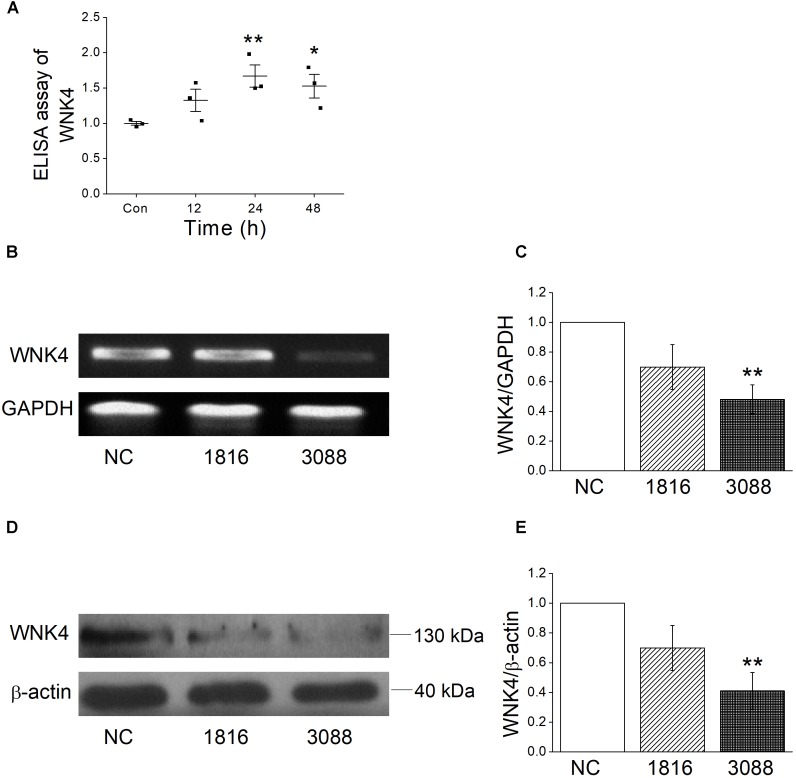
Influenza virus increases the intracellular WNK4 expression. **(A)** ELISA assay for WNK4, after treated with influenza virus (Flu). ^∗^*P* < 0.05, ^∗∗^*P* < 0.01, compared with control (Con), *n* = 3. **(B,C)** Representative reverse transcription PCR bands and statistic data for WNK4 knockdown, quantified using gray analysis (WNK4/GAPDH), after treated with siRNA WNK4-1816 and WNK4-3088. ^∗∗^*P* < 0.01, compared with negative control (NC), *n* = 3. **(D,E)** Representative western blot bands and statistic data for WNK4 knockdown. ^∗∗^*P* < 0.01, compared with negative control, *n* = 3. Data was presented as mean ± SE. One-way ANOVA followed by Bonferroni’s test was used to analyze the difference of the means for significance.

The above experiments supported that ENaC participate the regulation of influenza virus infected airway fluid, but the mechanisms of how influenza virus reduce ENaC activity were not very clear. WNK4 has been proved to be a protein kinase that regulates a variety of ion channels, including ENaC (Hadchouel et al., 2016; Castañeda-Bueno et al., 2017). We measured the intracellular WNK4 concentration by ELISA assay accordingly and as shown in Figure 6A, after MTECs were infected by influenza virus for 12 h, the concentration of WNK4 began to increase, and after 24 ∼ 48 h administration, WNK4 level elevated obviously, which had a significant difference compared with the control group (*P* < 0.01, *n* = 3).

### WNK4 Knockdown Rescued the Impact of Influenza Virus on ENaC

To further identify that influenza virus regulates ENaC activity through the pathway of WNK4, we applied siRNA-specific technology to knockdown WNK4 and test the efficiency first. Both reverse transcription PCR and western blot results showed that the knockdown efficiency of WNK4-3088 was better than that of WNK4-1816 (Figures 6B–E). Accordingly, we selected WNK4-3088 for the following WNK4 knockdown related experiments. The WNK4 antibody from the same company in our experiment had been used previously (Fang et al., 2016). The specificity of this antibody was confirmed by the company, using the transfected and non-transfected cells. The full length blot of WNK4 was shown in Supplementary Figure 2, and the 130 kDa band we chose for analysis was verified by the molecular weight information in the UniProt Knowledgebase.

**FIGURE 7:**
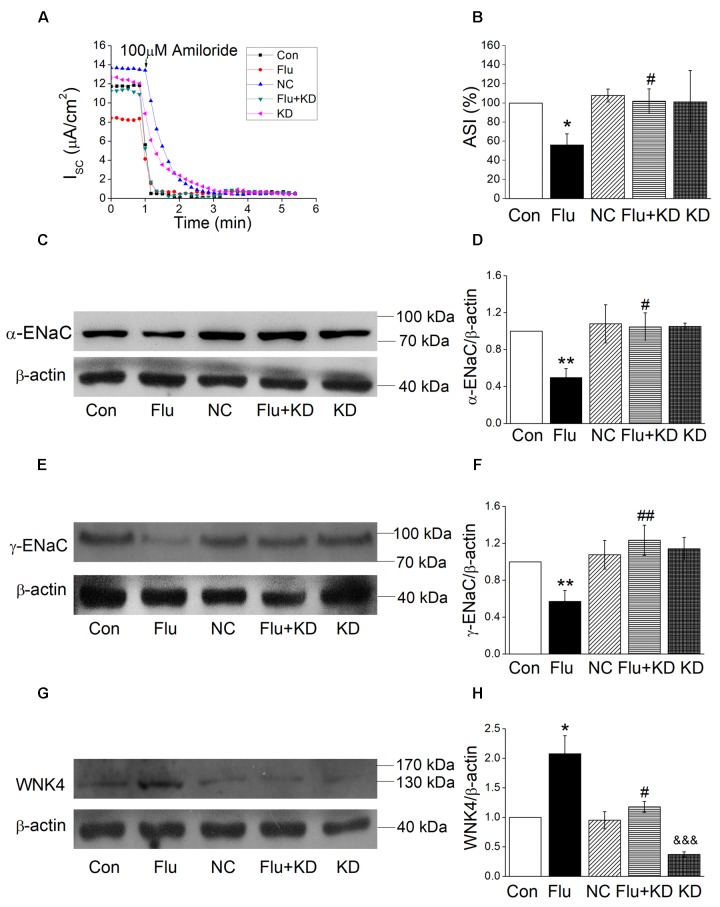
WNK4 knockdown rescued the impact of influenza virus on ENaC. **(A)** Representative Isc trace of control (Con), influenza virus infection (Flu), negative control (NC), WNK4 knockdown plus influenza virus infection (Flu + KD), and WNK4 knockdown (KD) group, respectively. **(B)** WNK4 knockdown relieves the inhibition of ASI by influenza virus. ASI of control was set as 100%. ^∗^*P* < 0.05, compared with control (Con), ^#^*P* < 0.05, compared with influenza virus infection group (Flu), *n* = 5. **(C,D)** Representative western blot bands and statistic data of WNK4 knockdown relieving the decreased α-ENaC expression by influenza virus. ^∗∗^*P* < 0.01, compared with control (Con), ^#^*P* < 0.05, compared with influenza virus infection group (Flu), *n* = 3. **(E,F)** Representative western blot bands and statistic data of WNK4 knockdown relieving the decreased γ-ENaC expression by influenza virus. ^∗∗^*P* < 0.01, compared with control (Con). ^##^*P* < 0.01, compared with influenza virus infection group (Flu), *n* = 4. **(G,H)** Representative western blot bands and statistic data of the WNK4 expression. ^∗^*P* < 0.05, compared with control (Con). ^#^*P* < 0.05, compared with influenza virus infection group (Flu), ^&&&^*P* < 0.001, compared with negative control (NC), *n* = 3. Data was presented as mean ± SE. One-way ANOVA followed by Bonferroni’s test was used to analyze the difference of the means for significance.

In our preliminary experiment, we compared the influenza virus infection with or without negative control for the siRNA approach, which showed no difference either in protein expression or short-circuit currents (Supplementary Figure 3). Accordingly, we chose the influenza virus infection group for the comparison with WNK4 knockdown plus influenza virus infection group. As shown in Figures 7A,B, WNK4 knockdown plus influenza virus infection group had a significantly greater ASI compared with the influenza virus infection alone (*P* < 0.05, *n* = 5), while the current of WNK4 knockdown and negative control had no difference with that of control group. We further detected the protein expression of ENaC and found that the decreased ENaC protein levels via influenza infection were relieved by WNK4 knockdown (Figures 7C–F). There are significant differences between the influenza virus infection group and the WNK4 knockdown plus influenza virus infection group (*P* < 0.01 ∼ 0.05, *n* = 3). Finally, we repeated western blot assay by examining the WNK4 expression and as expected, the protein expression level of WNK4 in influenza virus infection group was higher than that of the control group and WNK4 knockdown could reduce the enhanced expression of WNK4 caused by influenza virus infection (Figures 7G,H, *P* < 0.05, *n* = 3). These results suggested that influenza virus may decrease ENaC activity via WNK4 pathway.

## Discussion

There are numerous studies about the pathogenicity of influenza viruses, most of which focused on the inflammatory reactions that result from viral infections. In this paper, we studied the roles of tight epithelial cells of the airway and ASL in influenza virus invasion. Previous studies have shown that disorder of ASL homeostasis can lead to increasing risk of bacterial infection (Shah et al., 2016). Much evidence confirmed that Na^+^ absorption is an important means to maintain ASL homeostasis, and ENaC play a key role in Na^+^ absorption (Tarran et al., 2001; Ji et al., 2012). Studies have shown that ENaC play a major role in respiratory diseases (Huppmann et al., 2011; Almaca et al., 2013), thus we explored the effects of influenza virus on ENaC.

Our results showed that influenza viruses can reduce ENaC activity. MTECs were isolated from the trachea of the mouse and cultured through the model of the air–liquid interface, mimicking the physiological state of the normal tracheal epithelial cells, mainly consisting of goblet cells and cilia cells. Complying with the morphological and physiological characteristics of respiratory endothelial cells, it is a good cell model to study respiratory diseases, which has been applied in toxicology, respiratory tract infection, ions transport, cell carcinogenesis, and other experiments (Davidson et al., 2000; Horani et al., 2013). When establishing the air–liquid interface, cells begin to differentiate and start three-dimensional growth, with the decrease in trans-epithelial electrical resistance (You et al., 2002). And the transmission electron microscopy results suggested that influenza virus infection for 24 h didn’t disrupt the ultrastructure of MTECs, eliminating the impact of the tight junctions on ASI. We also measured the trans-epithelial electrical resistance in MTECs after influenza virus infection. Excluding these factors, we concluded that influenza virus could decrease ASI and inhibit Na^+^ transport without affecting tight junctions. Referring to the experimental results from Taryn Waugh (Waugh et al., 2013), we applied the MOI 0.6 virus dosage in our study and undoubtly found that the influenza virus could reduce ENaC expressions at the protein and mRNA level, respectively.

Followingly we measured the ASL height, during which we used FITC-dextran to label ASL, and the molecular weight of dyes in previous studies was not uniform. In order to get a more accurate result, we chose the small molecular weight dye to label the ASL, which could reach closer to the cell surface (Worthington and Tarran, 2011). In the fluorescence recovery after photobleaching experiment, we believed that the 2 μm below the upper ASL was the mucus layer. In normal MTECs, ASL height is 8.6 μm or so, and preciliary liquid height is about 5 μm, so the position of 2 μm below the upper ASL should be the middle position of mucus layer. Our transmission electron microscopy results showed that the length of the cilia in our cultured MTECs was approximate 5 μm, and preciliary liquid height of normal MTECs was equal to the length of cilia (Liu et al., 2013). Then we did the photobleaching at 2 μm below the upper ASL.

Previous studies have found that influenza viruses could activate the immune system, macrophage synthesis of nitric oxide synthase and tumor necrosis factor α (Kunzelmann et al., 2000). WNK kinase belongs to the silk/threonine family and its functional structure domain 2nd subunit and ATP-bound lysine residues are substituted by the cysteine residues. It has been found that the WNK kinase has four family members, WNKl–4, among which WNK4 can be activated by PKC-like kinases. There are more studies of WNK4 in the kidney disease, but the study of WNK4 in the respiratory system is rarely reported. It has been reported that while influenza virus contacts with the membrane surface, it will activate phospholipase C causing inositol triphosphate and diacylglycerol released, which then release the intracellular calcium and activate PKC (Hartshorn et al., 1990). WNK4 can regulate the activity of many ion channels, including ENaC. As a PKC downstream, we speculated that WNK4 would be activated to regulate ENaC activity via influenza infection. Our experiment results confirmed that the WNK4 concentration increased in influenza virus infected MTECs, further supported by the increased protein expression level of WNK4. Finally, we applied the siRNA-specific knockdown of WNK4 gene and found that WNK4 knockdown could enhance the decreased ENaC activity via influenza virus infection, demonstrating that influenza viruses reduce ENaC activity through intracellular WNK4.

## Conclusion

Influenza virus impairs the fluid transport in airway by disturbing ENaC function via WNK4 pathway.

## Author Contributions

HN and H-LJ conceived and designed the study. YH, ZZ, YD, and YC performed the study. HL, ZZ, and HZ analyzed the data. YH and HN drafted the manuscript. H-LJ revised the draft of manuscript. All authors corrected and approved the final version of the manuscript.

## Conflict of Interest Statement

The authors declare that the research was conducted in the absence of any commercial or financial relationships that could be construed as a potential conflict of interest.

## Abbreviations

ASI: amiloride-sensitive short-circuit currents
ASL: airway surface liquid
ENaC: epithelial sodium channels
Isc: short-circuit currents
MOI: multiplicity of infection
MTECs: mouse tracheal epithelial cells
PCR: polymerase chain reaction
WNK4: with-no-lysine-kinase-4
