# Supplementary material for: Upregulation of the WNK4 Signaling Pathway Inhibits Epithelial Sodium Channels of Mouse Tracheal Epithelial Cells After Influenza A Infection
Source: Front Pharmacol. 2019 Jan 22;10:12. doi: 10.3389/fphar.2019.00012 (PMC6349759; doi:10.3389/fphar.2019.00012)

Upregulation of the WNK4 signaling pathway inhibits epithelial sodium channels of mouse tracheal epithelial cells after influenza A infection

Yapeng Hou[1], Yong Cui[2], Zhiyu Zhou[1], Hongfei Liu[1], Honglei Zhang[1], Yan Ding[1], Hongguang Nie[1,*], Hong-Long Ji[3,4]

[1]Department of Stem Cells and Regenerative Medicine, Key Laboratory of Cell Biology, National Health Commission of China, and Key Laboratory of Medical Cell Biology, Ministry of Education of China, China Medical University, Shenyang, China; [2]Departments of Anesthesiology, the First Affiliated Hospital of China Medical University, Shenyang, China; [3]Department of Cellular and Molecular Biology, University of Texas Health Science Center at Tyler, Tyler Texas; [4]Texas Lung Injury Institute, University of Texas Health Northeast, Tyler Texas, USA

* Corresponding author:

Hongguang Nie, hgnie@cmu.edu.cn

Supplementary Figure 1. Western blot of α- and γ-ENaC proteins in MTECs. Blots were incubated with specific antibodies against α- and γ-ENaC subunits (*left panels*, A-B). To demonstrate specificity, the blots were incubated with ENaC antibodies in the presence of an excess of the corresponding immunizing peptides (*right panels*, A-B). Molecular weights of all bands have been indicated on the right of the blot. The specific bands about 80 kDa and 95 kDa for α- and γ-ENaC proteins could be seen, according to the manufacturer's manual, respectively.

Supplementary Figure 2. The full-length blots/gels of WNK4 protein extracted from MTECs. Molecular weights of all bands have been indicated on the right of the blot. The specific band about 130 kDa for WNK4 protein could be seen, according to the manufacturer's manual.

Supplementary Figure 3. Negative control for WNK4 knockdown didn't affect the impact of influenza virus on ENaC. (A) Representative Isc trace after MTECs were infected with influenza virus (Flu) and influenza virus plus negative control (Flu + NC), then 100 μM amiloride was applied. (B) Statistic ASI in MTEC monolayers. ASI was defined as the difference between the total current and the amiloride-resistant current and the ASI in Flu group was set as 100%, n = 4. (C-F) Representative western blot bands and statistic data of α- and γ-ENaC protein expression by influenza virus (Flu) and influenza virus plus negative control (Flu + NC). n = 3-4. Data was presented as mean ± S.E. Student's-t-test was used to analyze the difference of the means for significance. NS, $P > 0.05$, compared with influenza virus group (Flu).

Supplementary Figure 1

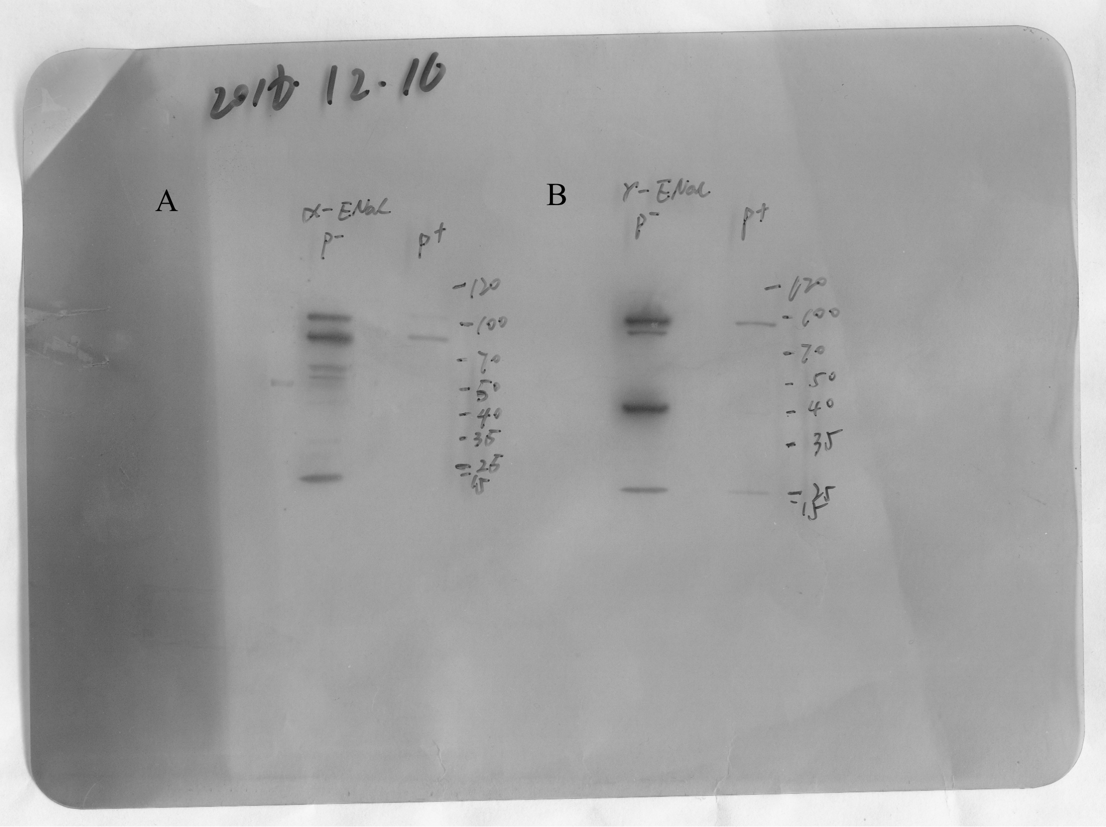

Supplementary Figure 2

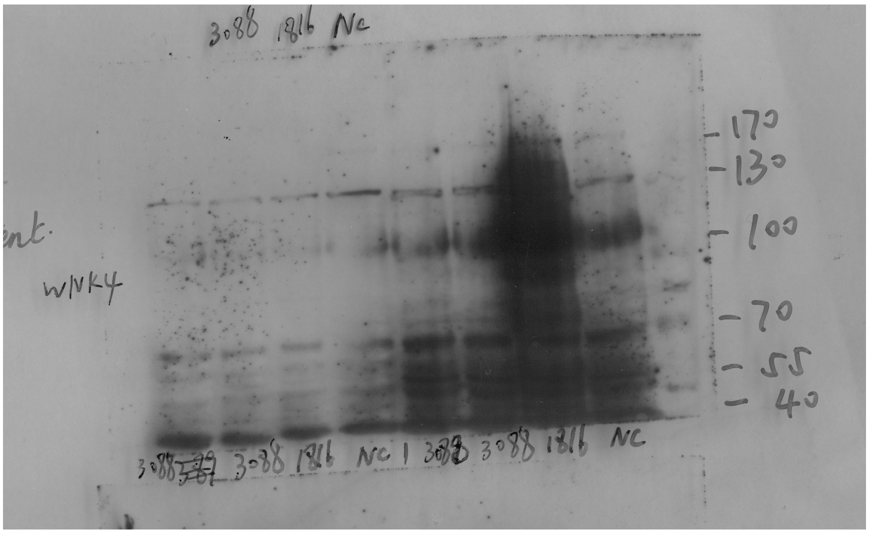

Supplementary Figure 3

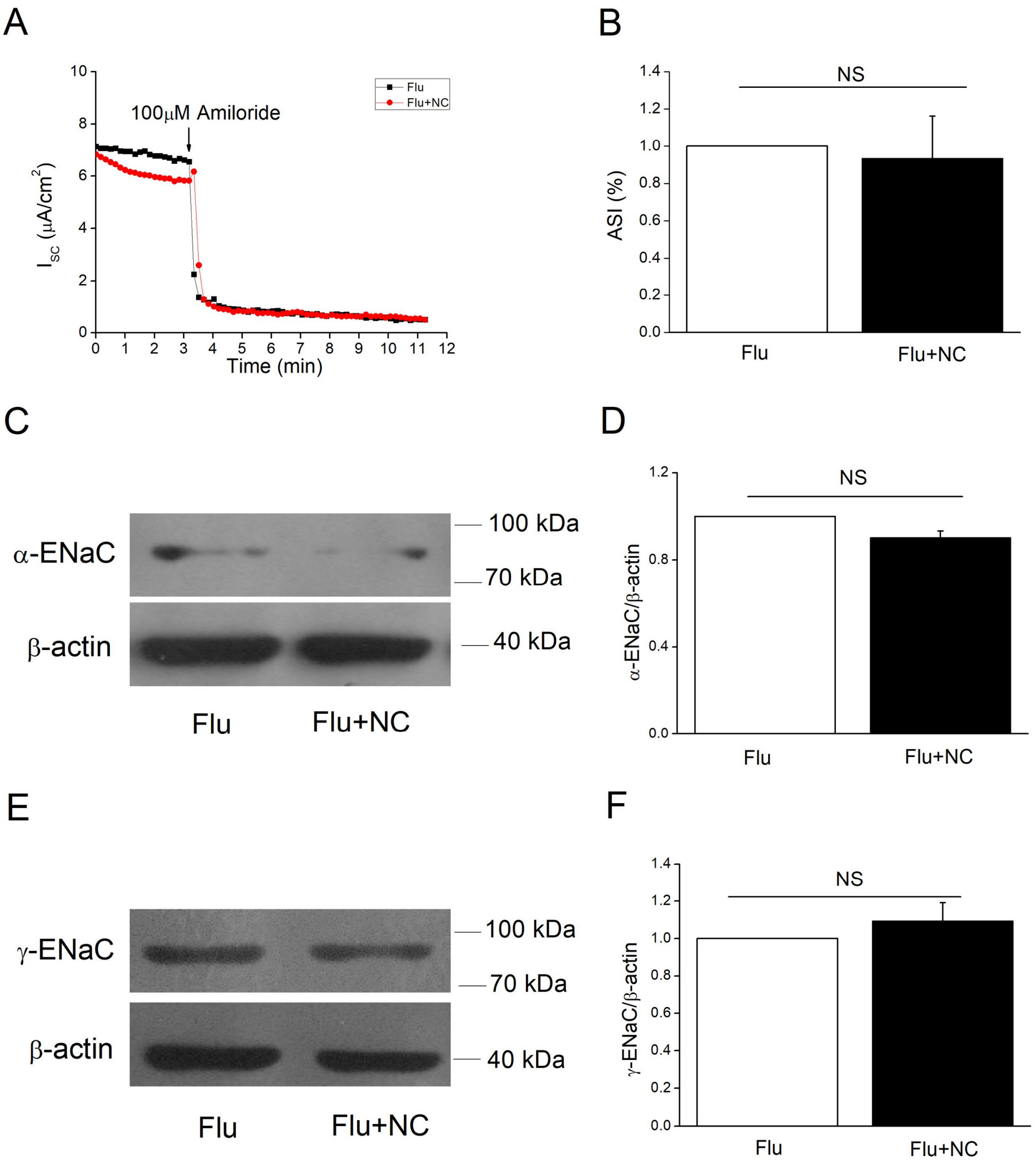

Supplement: Supplementary file 1 [file Data_Sheet_1.PDF]
